# How elevated CO_2_ affects our nutrition in rice, and how we can deal with it

**DOI:** 10.1371/journal.pone.0212840

**Published:** 2019-03-05

**Authors:** Kazuhiro Ujiie, Ken Ishimaru, Naoki Hirotsu, Seiji Nagasaka, Yuichi Miyakoshi, Masako Ota, Takeshi Tokida, Hidemitsu Sakai, Yasuhiro Usui, Keisuke Ono, Kazuhiko Kobayashi, Hiroshi Nakano, Satoshi Yoshinaga, Takayuki Kashiwagi, Jun Magoshi

**Affiliations:** 1 Shimane University, Matsue, Shimane, Japan; 2 Institute of Crop Sciences, NARO, Tsukuba, Ibaraki, Japan; 3 Toyo University, Itakura-machi, Gunma, Japan; 4 Institute for Agro-Environmental Sciences, NARO, Tsukuba, Ibaraki, Japan; 5 Hokkaido Agricultural Research Center, NARO, Hokkaido, Japan; 6 Graduate School of Agricultural and Life Sciences, The University of Tokyo, Bunkyo-ku, Tokyo, Japan; 7 Kyushu Okinawa Agricultural Research Center, NARO, Fukuoka, Japan; 8 Central region Agriculture Research Center, NARO, Tsukuba, Ibaraki, Japan; 9 Utsunomiya University, Utsunomiya, Tochigi, Japan; INRA, FRANCE

## Abstract

Increased concentrations of atmospheric CO_2_ are predicted to reduce the content of essential elements such as protein, zinc, and iron in C_3_ grains and legumes, threatening the nutrition of billions of people in the next 50 years. However, this prediction has mostly been limited to grain crops, and moreover, we have little information about either the underlying mechanism or an effective intervention to mitigate these reductions. Here, we present a broader picture of the reductions in elemental content among crops grown under elevated CO_2_ concentration. By using a new approach, flow analysis of elements, we show that lower absorption and/or translocation to grains is a key factor underlying such elemental changes. On the basis of these findings, we propose two effective interventions—namely, growing C_4_ instead of C_3_ crops, and genetic improvements—to minimize the elemental changes in crops, and thereby avoid an impairment of human nutrition under conditions of elevated CO_2_.

## Introduction

On the basis of the results of free-air CO_2_ enrichment (FACE) experiments, Myers et al. [1, 2] argued that increasing CO_2_ concentration ([CO_2_]) over the next 40–60 years would lead to deficiencies of essential elements, including nitrogen (protein), zinc, and iron, in C_3_ grains and legumes. In turn, this would threaten human nutrition, especially in low-resource countries where people depend on grain crops for their micronutrient intake. Studies supporting this prediction have been reported since the 1990s, including meta-analyses [3, 4, 5] and FACE experiments [6, 7]. C_3_ grains and legumes supply 60% of dietary Zn and/or Fe for roughly 2 billion people [1]. Among these crops, rice is an important staple for half of the world’s population and also supplies more than 30% of total protein for 3.4 hundred million individuals [8].

Elemental content varies among organs in a plant. For example, the elemental content differs markedly between brown rice and the “polished” grains that we ordinarily consume. To our knowledge, all previous studies except one [9, 10] have analyzed the mineral content of brown grains. In many countries, the rice plant body (“straw”) is supplied as feed to cattle [11]. It is not known whether the response of the elemental content to elevated [CO_2_] is the same in brown grains as it is in polished rice or the plant body; if not, predictions of deficiencies might not reflect the full scale of damage to the nutritional qualities of rice caused by elevated [CO_2_].

More importantly, the information available on the effect of [CO_2_] on grain crops is limited, and little is known about either the underlying mechanism or practicable strategies to avert a future nutritional crisis. Here we explore three points concerning the potential damage to human nutrition caused by elevated [CO_2_]. First, we present a broader picture of its impact, including data for the plant body of rice and other crops (e.g., feed crops and leaf vegetables); second, we investigate the physiological mechanism underlying the reduction in elemental content; and third, we propose feasible targets to minimize the effect of elevated [CO_2_] on the basis of this mechanism.

## Materials and methods

### Plant materials

**Fig 1:**
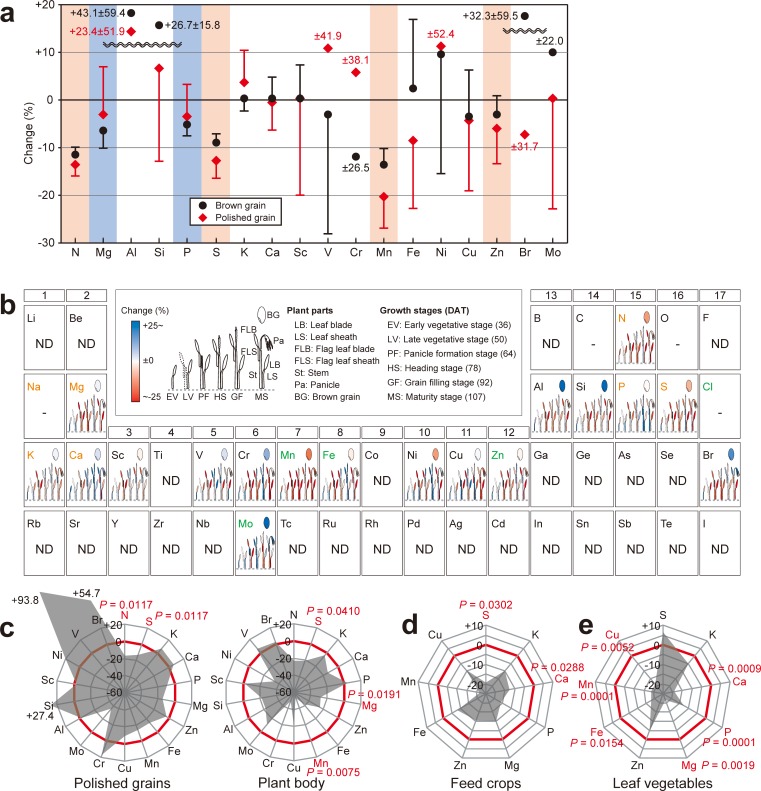
Influence of elevated [CO_2_] on elemental content in crops. (a) Comparison of changes in the content of all tested elements under elevated [CO_2_] between brown rice (black circles) and polished grains (red diamonds). Data are presented as the mean and one side of 95% confidence intervals (bars) of nine varieties. Red and blue backgrounds denote a significant difference (*P* < 0.05), where previous work on brown grain has under- or overestimated, respectively, elemental changes in polished grains. For Al, Si, and Br, the values were off the scale of the *y* axis, as indicated by the wavy line. (b) Time-dependent changes in the elemental content of each part of the rice plant under elevated [CO_2_]. Data are presented as the mean. (c) Percentage change in the elemental content of polished grains and plant body (“straw”) of rice (“*Koshihikari*”). (d) Estimated change in elemental content in feed crops under elevated [CO_2_]. (e) Estimated change in elemental content in leaf vegetables under elevated [CO_2_]. The raw data are provided in Table 1 and S4 Table. In (c)-(e), essential elements are arranged in decreasing order of intake requirement. Elements that decreased significantly (*P* < 0.05) under elevated [CO_2_] are shown in red.

Rice (*Oryza sativa* L.) was grown under FACE conditions in Tsukubamirai, Japan, from 2010 to 2012 [12]. In 2010, nine rice varieties (“*Koshihikari*”; japonica “*Nipponbare*”; japonica high-yield “*Hoshiaoba*”; large grain type “*Bekoaoba*”; indica high-yield “*Takanari*”, “*Hokuriku 193*”, and “*IR72*”; tropical japonica “*Lemont*”; and japonica-dominant high-yield “*Momiroman*”) as well as a “*Koshihikari*” line in which part of chromosome 5 was substituted by “*Kasalath*” (SL-rg5) [13] were cultivated. We also grew “*Koshihikari*” and a chromosome segment substitution line for *GS3*25 (SL-GS3) in the genetic background of “*Koshihikari*” in 2012. Seeds of the two chromosome segment substitution lines have been deposited in the Rice Genome Resource Center (http://www.rgrc.dna.affrc.go.jp/index.html). The panicles were harvested at maturity and the brown and/or polished grains were analyzed for elemental content. For the analysis of elemental stocks and flows, three tillers of “Koshihikari” were sampled from three plants for each replication at six stages of growth in 2010. Sampling was conducted at the early vegetative (36 days after transplanting [DAT]), late vegetative (50 DAT), panicle formation (64 DAT), heading (78 DAT), grain filling (92 DAT), and maturity (107 DAT) stages. These samples were divided into six parts (see Fig 1B), and dried portions were powdered and used for elemental measurements. In 2011, the flag leaves of “Koshihikari” were harvested at the heading stage and immediately stored in liquid nitrogen. These leaves were used in microarray analysis and real-time PCR. In addition, we grew three feed crops (*Avena sativa*, *Lolium perenne*, and *Medicago sativa*) and four leaf vegetables (*Brassica rapa* var. *perviridis* [2 cultivars], *Brassica rapa* var. *chinensis*, *Brassica oleracea* var. *capitata*, and *Beta vulgaris* var. *cilia*) at two concentrations of CO_2_ in semi-closed growth chambers [14]. The CO_2_ concentrations were fixed at 370 (ambient) and 700 ppm (elevated). The above-ground parts of these plants were sampled 30 days after sowing, except for *Lolium perenne* and *Medicago sativa*, which were sampled at 40 days.

### Measurement of elemental content

The content of nitrogen and other elements was analyzed by, respectively, an NC analyzer (SUMIGRAPH NCH-22, Sumika Chemical Analysis Service) and an energy-dispersive X-ray fluorescence spectrometer (EDXRF, element analyzer JSX-3201, JEOL). For the analysis of all elements except nitrogen, three 13-mm-diameter tablets were formed from each replicate, and each tablet was measured three times according to the method of Kashiwagi et al. (2009) [15]. The average of these nine measurements was taken as the result for each replicate. Differences in mineral content between different [CO_2_] conditions were tested by Z-test.

### Calculation of elemental stocks and flows

**Fig 2:**
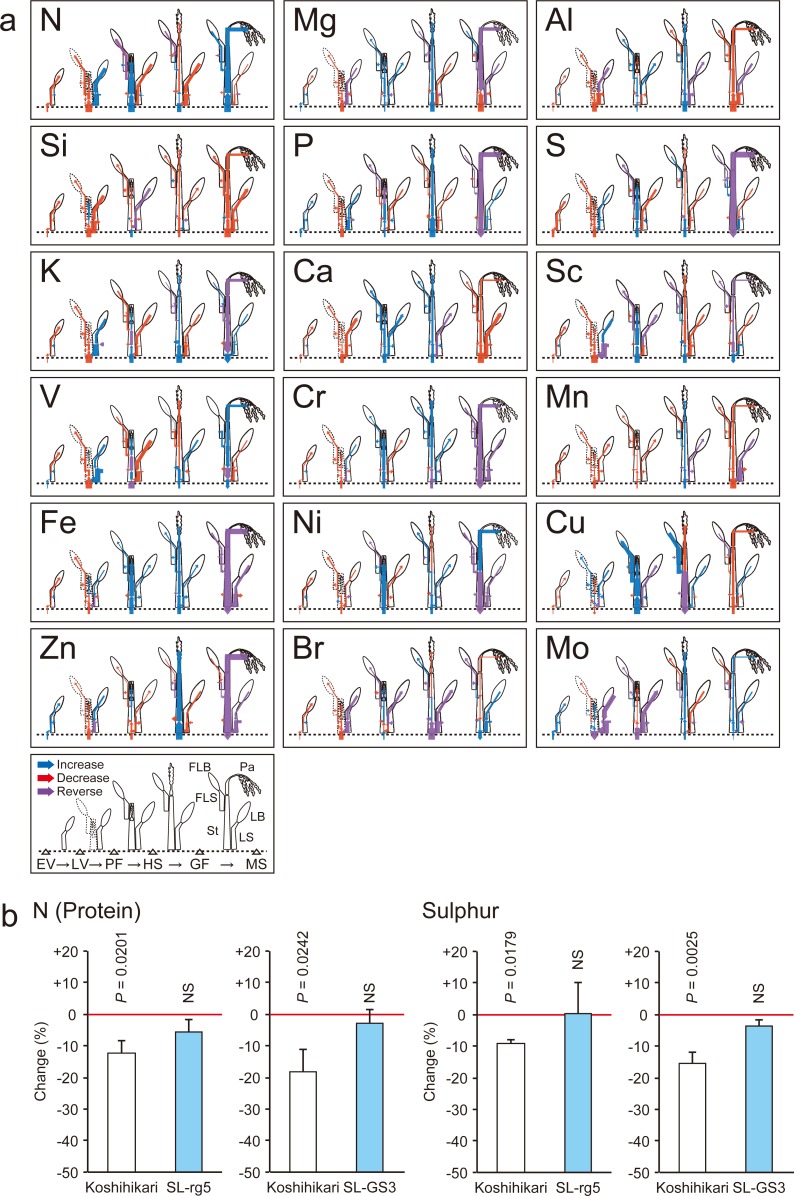
Influence of elevated [CO_2_] on flow of elements in a rice plant during growth, and genetic improvement to prevent elemental reduction in grains. (a) Change in elemental flow in rice (“*Koshihikari*”) under elevated [CO_2_] as compared with ambient [CO_2_]. Arrows indicate the direction of elemental flow: blue shows an increase, red a decrease, and grey no change under elevated [CO_2_] versus ambient [CO_2_]. Purple arrows indicate that the direction of flow was reversed by CO_2_ treatment. The extent of the change in flow is indicated by arrow width; the part of the plant showing the greatest changes for each element is represented by the widest arrow. Abbreviations are defined in Fig 1B. (b) Percentage change in the nitrogen (protein) and sulfur content of grains of wild-type “*Koshihikari”* and a chromosomal segment substitution line containing *rg5*18 or *GS3*19 grown under elevated [CO_2_] relative to ambient [CO_2_].

The relative stock of each element in the plant was calculated as a product of the relative content and dry weight. The relative rate of elemental translocation between plant parts during different growth stages, which we call “flow,” was estimated by using the stock of the element. Flow was calculated as follows: (1) Flow = (C_2_W_2_—C1W1)/(t_2_—t_1_), where C and W are the relative content and dry weight, respectively; C_n_W_n_ is the stock value of the element; C_2_W_2_—C_1_W_1_ indicates the change in the content of the element between growth stages; and t_2_—t_1_ is the number of days between the growth stages. A positive flow value indicates that the element has flowed into the plant tissue. Conversely, a negative value means the element has translocated to other tissues or has been discharged by defoliation. The flow of elements among organs was estimated by net flows for each element, as described for the modeling of carbon and nitrogen flows [16, 17]. Hierarchic clustering of the flow pattern was conducted by a group average method based on Euclidean distance. In Fig 2A, we show a representation of the difference in net flows between ambient and elevated [CO_2_] conditions.

### Estimation of the impact on feed crops and leaf vegetables

Changes in element content were estimated from data for eight feed crops and seven leaf vegetables, including eight new data sets obtained in this study (feed crops: *Avena sativa*, *Lolium perenne*, *Medicago sativa*, and rice straw; leaf vegetables: *Brassica rapa* var. *perviridis* [two cultivars], *Brassica rapa* var. *chinensis*, *Brassica oleracea* var. *capitata*, and *Beta vulgaris* var. *cilia*). Other data were taken from six previous reports (see legends of Table 1 and S4 Table). The effects of elevated [CO_2_] on elemental content were assessed by Z-test. Elements that were not measured were excluded from the statistical analysis.

**Table 1:** Effect of elevated [CO_2_] on elemental content in feed crops. ND means no data. *Significant at P<0.05. The values mean the ratio of elemental content under elevated [CO_2_] to content under ambient condition (%).

Plant species	Plant type	S	K	Ca	P	Mg	Zn	Fe	Mn	Cu	Reference
*Avena sativa*	C3	56.21	82.19	101.90	41.76	95.95	57.17	66.28	54.12	56.80	new data
*Lotium perenne*	C3	88.17	105.35	97.01	105.82	92.93	87.74	105.56	62.98	64.22	new data
*Oryza sativa* (straw)	C3	79.49	96.19	86.02	104.11	71.88	75.27	58.53	47.23	77.97	new data
*Bromus tectorum*	C3	ND	82.35	83.87	ND	ND	ND	ND	84.21	ND	Blank et al. [35]
*Festuca vivipara*	C3	ND	62.16	54.77	52.04	62.98	ND	ND	ND	ND	Baxter et al. [36]
*Medicago sativa*	C4	98.31	88.72	98.94	96.53	116.74	89.54	86.58	146.16	96.14	new data
*Bouteloua curtipendula*	C4	ND	94.03	101.58	88.18	87.57	126.36	108.09	73.98	141.37	Polley et al. [37]
*Sorghastrum nutans*	C4	ND	109.82	74.44	99.71	101.21	101.25	125.19	77.74	108.38	Polley et al. [37]
Ave.		80.54	90.10	87.32	84.02	89.89	89.55	91.70	78.06	90.81	
*P* value (ZTEST)		0.0302	0.0623	0.0288	0.1061	0.1380	0.2750	0.4327	0.0765	0.4733	
		*	NS	*	NS	NS	NS	NS	NS	NS	
Ave in C3		74.62	85.65	84.71	75.93	80.93	73.39	76.79	62.14	66.33	
Ave in C4		98.31	97.53	91.65	94.80	101.84	105.71	106.62	99.30	115.30	

### Gene expression analyses

Total RNA was extracted from a rice flag leaf at the heading stage. We used a rice genome 44K oligonucleotide microarray (G2519F#15241, Agilent Technologies) containing approximately 42,000 oligonucleotides synthesized on the basis of nucleotide sequences and full-length cDNA data from RAP-DB [18, 19]. Fluorescence probe labeling, hybridization, scanning, and data analysis were performed according to the manufacturer’s instructions. We focused only on expression levels that showed a more than 1.5-fold change in expression that was statistically significant (*P* < 0.05 by Significance Analysis of Microarray). The extracted total RNA was converted into first-strand cDNA, and then quantitative RT-PCR was performed with the same samples used in the microarray. *Actin* (*Act 1*) was used as a control. The following primers were used: for *Act1*, forward GACTCTGGTGATGGTGTCAGC, reverse GGCTGGAAGAGGACCTCAGG; for *OsZIP5*, forward CTGGAGCTGGGAGTGGTGGT, reverse ATGTCGACGAGCGCCATGTA; for *OsNAS3*, forward GTGATCAACTCCGTCATCATC, reverse TCAGTCTCATCATGGGAAAAA.

## Results

### Influence of elevated [CO_2_] on elemental content in rice

As compared with ambient conditions, elevated [CO_2_] reduced the content of N (protein), S, Mn, and Zn in polished grains by 13.5%, 12.6%, 20.5%, and 5.9%, respectively (Fig 1A). The corresponding decreases in brown rice were, respectively, 2.0%, 3.5%, 7.0%, and 3.0% smaller than those in polished grains. For both types of grain, the decrease in Fe content was not significant; however, polishing reduced Fe content by 10.9%. The influence of elevated [CO_2_] varied among plant parts and growth stages (Fig 1B). As compared with the grains, the plant body exhibited a greater decrease in elemental content under elevated [CO_2_] (Fig 1C): for example, the content of Mg, S, and Mn in the plant body decreased by 28%, 21%, and 53%, respectively (all *P* < 0.05), which was approximately 2-, 1.5-, and 4-fold greater than the decreases observed in grains.

### Influence of elevated [CO_2_] on elemental content in feed crops and leaf vegetables

The analysis of eight feed crops, which combined our new data including rice straw with data from three previous studies (Table 1), showed that elevated [CO_2_] was associated with a significant decrease in five major and three minor essential minerals (Fig 1D). Further, the content of Ca and S was reduced by 12.7% and 19.5%, respectively (all *P* < 0.05; Fig 1D, Table 1). In contrast, as compared with C_3_ feed crops, the elemental content of the plant body of C_4_ crops was little affected by elevated [CO_2_]. The analysis of seven leaf vegetables, again combining previous data with our current results, indicated that elevated [CO_2_] was associated with a reduction of about 20% in the concentration of multiple essential minerals, including Mg, P, Ca, Mn, Fe, and Zn (Fig 1E).

### Influence of elevated [CO_2_] on the expression of transporter genes

Gene expression profiles were assessed in a flag leaf of rice obtained around the heading stage. As compared with ambient conditions, 798 genes were significantly down-regulated by at least one-third, and the expression of 529 genes increased 1.5-fold under FACE conditions. Among the genes showing changes, 14 encoded transporters. Ten of these genes, including K and heavy metal transporters, were down-regulated, whereas the expression of the other four increased (Table 2 and S1 Fig). Among the ten down-regulated genes, five have functions in the import of minerals into cells without the involvement of ABC transporters.

**Table 2:** List of genes for element transport that is affected by elevated [CO_2_].

Element	CODE	GENE	Fold-change	P value (SAM)
Fe	Os07g0689600	OsNAS3	2.66	0.0317
Various element	Os09g0333500	PDR-like ABC transporter (PDR3 ABC transporter).	2.14	0.0163
K	Os04g0445000	Potassium channel SKOR (Stelar K(+) outward rectifying channel).	1.64	0.0273
Various element	Os06g0695800	ABC transporter related domain containing protein. (potassium transport)	1.58	0.0306
S	Os01g0719300	Sulfate transporter 3.1 (AST12) (AtST1). (absorption)	0.64	0.0111
Various element	Os09g0472200	ABC transporter.	0.60	0.0225
Mg	Os03g0137700	Mg2+ transporter protein, CorA-like family protein. (absorption)	0.59	0.0034
Zn	Os03g0411800	Zinc transporter 11 precursor (ZRT/IRT-like protein 11)	0.57	0.0021
Heavy metal	Os04g0464100	Heavy metal transport/detoxification protein domain ontaining protein. (absorption/desorption)	0.53	0.0373
Fe, Zn	Os04g0613000	Zinc transporter 1 precursor (ZRT/IRT-like protein 1). *OsZIP3*	0.53	0.0487
Various element	Os11g0155600	ABC transporter related domain containing protein.	0.51	0.0014
Heavy metal	Os02g0582600	Heavy metal transport/detoxification protein domain containing protein.	0.47	0.0037
Mn, Fe, Cu, Zn	Os05g0472700	Zinc transporter protein *OsZIP5*.	0.46	0.0003
K	Os08g0466200	K+ potassium transporter family protein. (absorption)	0.35	0.0076

### Flow analysis of elements in rice throughout different growth stages

To clarify the physiological factors responsible for the elemental changes, we analyzed the influence of elevated [CO_2_] on the elemental flow in rice. Retranslocation of N, S, and Cu from leaf blades was lower under elevated [CO_2_] than under ambient [CO_2_] conditions during the maturity stage (Fig 2A). Elements were clustered into three broad groups according to their flow pattern under ambient [CO_2_] conditions (increased, decreased, or reversed), and notably, Cu, Mo, and Mn moved into a different group under FACE conditions (S2 Fig). The large change in flow pattern indicates a shift in the allocation of elements among tissues.

## Discussion

As a first step towards mitigating the damage caused by elevated [CO_2_], we require a complete picture of the reduction in the elemental content of crops expected in the near future. As compared with polished grains, data from brown rice have underestimated the decrease in N (protein), S, Mn, and Zn under elevated [CO_2_] by 2.0%, 3.5%, 7.0% and 3.0%, respectively (Fig 1A). Our results suggest that increasing [CO_2_] levels might have more serious consequences than previous influential predictions that were based on brown rice [1]. In particular, the drop in N in polished rice would negatively affect the nutritional status of the 153 million individuals of Bangladesh, who depend largely on rice for their protein intake and are already estimated to have an individual daily protein intake below the standard of “hungry” as defined by FAO/WHO (S1 Table).

Although we used the same rice samples as in a previous work [1], we found different changes in several elements. For example, Myers et al. [1] found that Fe content was reduced by 5.2% under elevated [CO_2_], whereas we found a slight increase. This may be because the elemental content of rice grains can be affected by environmental factors, such as air temperature [20], during the ripening period. Among the 18 varieties that Myers et al. [1] used, we selected nine varieties with similar heading behavior that were appropriate for predicting damage to actual production due to FACE, although this narrowed the genetic diversity represented. This difference in aims between Myers et al.’s [1] and our study might also explain our different results.

Our results showed that elevated [CO_2_] might also impair human nutrition via leaf vegetables and feed crops that we either eat directly or utilize as animal feed, not only via grain crops. As compared to the grain, elemental content declined markedly in the plant body of rice. For example, Mg and Mn content in the plant body decreased 2- and 4-fold, respectively (Fig 1C). These data, and the difference between polished (endosperm) and brown (endosperm plus bran) grains demonstrate that the elemental reduction due to elevated [CO_2_], is specific to each organ. The impact on leaf vegetables and feed crops should also be noted, because these are important sources of micronutrients despite being consumed in smaller amounts than staple grains.

In the analysis of feed crops, which combined our new data including rice straw with data from three previous studies (see Fig 1D, Table 1), elevated [CO_2_] decreased the content of five major (S, K, P, Ca, and Mg) and three minor (Zn, Fe, and Mn) essential minerals by 8.3% to 21.9%. It is especially noteworthy that the content of Ca was reduced by 12.7%. At present, much of the world’s population is facing a deficiency of Ca [21, 22]. Approximately 25.4 million adults in the United Kingdom and the United States have a high risk of Ca deficiency [21], and 54% of the population of Africa (5.7 hundred million individuals) is at risk of Ca deficiency [22]. Leaf vegetables are an important source of essential minerals; for example, in the United Kingdom and the United States, 10% of Ca and Mg intake comes from leaf vegetables [7]. The main source of Ca is milk, however, with UK adults obtaining half of their Ca intake from milk and dairy products [7]. The elemental content of the livestock's ingested feed correlates with that of the milk that they produce [23, 24]; therefore, the lower elemental content of feed crops grown under elevated [CO_2_] is likely to reduce the elemental content of milk and dairy products. Ultimately, elevated [CO_2_] might affect human nutrition and health even in high-income countries, where food supplements for micronutrients including Ca are readily available.

The effect of elevated [CO_2_] on Mn and S content has not previously been a research focus because we generally obtain an adequate intake of these minerals in daily life [1–5]. Our present data showed that, under elevated [CO_2_], the content of these elements decreased markedly across various plant parts across several species (Fig 1). A previous study showed that consumption of a Mn-deficient diet (0.11 mg/day; about 3% of RDA) for 39 days led to a mild form of dermatitis among five of seven men [25]. Sulfur is present primarily as a constituent of S-containing amino acids; a restricted dietary supply of these acids slows the synthesis rate of whole-blood glutathione-SH, an important antioxidant, and diminishes turnover. Glutathione-SH deficiency is a risk factor for chronic liver disease [26]. In the next 40–60 years, therefore, we may face unexpected challenges to our health owing to a reduction in the intake of minerals that we currently obtain in sufficient amounts.

Our new approach, flow analysis of elements, suggested that lower absorption and/or translocation is a key factor underlying the lower elemental content, at least in rice grains, under elevated [CO_2_] (Fig 2A). For example, lower retranslocation of N and S from leaf blades during the maturity stage led to a reduction in these elements in grains under elevated [CO_2_]. During retranslocation, accumulated elements are transported with carbohydrates in phloem sap. These results therefore suggest that elevated [CO_2_] might affect elemental retranslocation via carbohydrate translocation. Zn- and Fe-regulated transporter-like protein 5 (OsZIP5) plays a main role in Zn deficiency and in the import of mineral ions such as Mn^2+^, Fe^2+^, and Cu^2+^, and is controlled at the transcriptional level in rice [27]. Under FACE conditions, its expression level was reduced by one-half, which might damage the distribution of these essential minerals (Table 2). In addition, Fe deficiency negatively regulates the expression of rice *nicotianamine synthase gene 3* (*OsNAS3*), which is related to transport [28]. Therefore, high [CO_2_] might affect the expression of related transporter genes and hence absorption and/or translocation of minerals.

On the basis of the mechanism underlying the reductions in the content of essential elements, there are two potentially effective interventions to maintain the nutritional levels of food sources grown under elevated [CO_2_]. In contrast to C_3_ crops, the elemental content of the grains in C_4_ crops such as maize and sorghum is reported to be little affected by elevated [CO_2_] [1]. Accordingly, we found that the elemental content in C_4_ plants was rarely reduced by elevated [CO_2_] not only in the grain but also in the plant body (Table 1). A similar tendency was reported by Loladze [4], who estimated the effect of elevated [CO_2_] on the mineral contents in various C_3_ and C_4_ plants in a large meta-analysis. Thus, to minimize the nutritional degradation caused by increasing [CO_2_], the first solution might be to cultivate C_4_ instead of C_3_ crops. This strategy would be effective for feed crops and for leaf vegetables where we consume the plant body. Because nutrient profiles and suitability for cultivation differ between C_3_ and C_4_ crops, C_3_ crops cannot be replaced in every situation. Under elevated [CO_2_], C_4_ plants decrease their transpiration rate, similar to C_3_ plants, but the parallel increase in both photosynthesis and carbohydrate content is greater in C_3_ plants [29]. The carbohydrates that increase under elevated [CO_2_] might further dilute elemental content (termed the “dilution effect”) [4]. As a result, the decrease in elemental content might be more severe in C_3_ plants than in C_4_ plants. Because we found that different elements were reduced at different rates, however, control mechanisms other than the dilution effect were probably operating.

The second potential intervention is based on the observation that, across C_3_ crops, elevated [CO_2_] significantly reduced the content of N and S, elements for which daily nutritional requirements are the highest [26]. The flow pattern of N and S suggests that reduced retranslocation to the panicle from stocks in the plant body at approximately the grain-filling stage is the main mechanism governing the observed reduction in these elements under high [CO_2_] (Fig 2A). N and S are retranslated with carbohydrate in sap [30, 31], and a greater sink (grain) size induces higher carbohydrate translocation [32]. We therefore hypothesized that improving carbohydrate translocation or expanding sink size might alleviate the elemental decline in grains grown under elevated [CO_2_]. We found that, under FACE conditions, the content of N (protein) and S significantly decreased in grains from a premium Japanese cultivar, “*Koshihikari*” (control), whereas N and S content was maintained in two “*Koshihikari*” lines containing a chromosomal segment substitution of *rg5*, a locus that improves carbohydrate translocation ability to grains [13], or substitution of *GS3*, a gene controlling grain size [33] (Fig 2B). These results show that improvement in retranslocation is a promising target to alleviate the elemental decline in grains grown under elevated [CO_2_]. By introducing a locus via molecular markers, it is possible to improve a single trait of a cultivar while maintaining other agronomic traits for several years [27, 34]. Combining such a breeding program with verification using FACE might yield new varieties to minimize the reduction of elements in grains of C_3_ crops under elevated [CO_2_].

In summary, we have shown that elevated [CO_2_] has the potential to cause damage to human nutrition and health via leaf vegetables and feed crops, not only grain crops. The greatest threat comes from the more severe drop in N and Zn in polished rice grains, and the lower content of Ca in feed crops and leaf vegetables. In addition, a reduction in the intake of minor minerals such as Mn and S that we currently obtain in sufficient amounts might cause unanticipated health risks. Flow analysis and transcriptomics suggested that lower absorption and/or translocation of elements is a key factor underlying the lower elemental content in rice grains, and the lower expression of related transporter genes under elevated [CO_2_] might also play a role. On the basis of this mechanism underlying the decrease in elemental content, we have proposed two practical strategies—the cultivation of suitable C_4_ instead of C_3_ crops and the genetic development of C_3_ grain crops with molecular markers—to avoid this impending crisis in human nutrition.

## Supporting information

S1 Table Countries whose populations received protein from rice in 2011. Source: United Nations Food and Agriculture Organization food balance sheets and 2011 United Nations estimated population. (http://faostat3.fao.org/browse/FB/*/E) Under elevated [CO2], countries shown on a red background were estimated the protein supplies at the below level of hunger. The standard of hunger (52.5g/day) is calculated by the below; Standard in cal. by WHO/FAO 2,100 kcal per a day (ref. 1) Standard in protein by UNHCR, UNICEF, WFP and WHO <10% of cal. (ref. 2) Cal. per protein 4 kcal/g.
(PDF)

S2 Table The mineral content of brown and polished grain in rice (Value per 100g). Source: USDA National Nutrient Database for Standard Reference (long and medium grain rice) (http://ods.od.nih.gov/). Standard tables of food composition in Japan, Fifth Revised and Enlarged Edition—2005 - (short grain rice). (http://www.mext.go.jp/b_menu/shingi/gijyutu/gijyutu3/toushin/05031802/002/001.pdf)
(PDF)

S3 Table The ratio in mineral content between brown and polished grain in rice. The values indicate the ratio of the mineral content in polished grain to content in brown grain (= 100).
(PDF)

S4 Table Analysis of elevated CO_2_ effects on elemental contents in leaf vegetables. ND means no data. ***, **, * Significant at P<0.001, 0.01, 0.05. In plant species, *B*. means *Brassica*.
(PDF)

S1 Fig Expression change of *OsNAS3* (Os07g0689600) and *OsZIP5* (Os05g0472700) under elevated [CO_2_] condition relative to ambient [CO_2_]. *Actin* (*Act1*) was used as the control. Data are presented as mean ± s.d. (n = 3). Student’s t tests were used to calculate P values.
(PDF)

S2 Fig Dendrogram for elemental flows in elevated [CO_2_] and ambient [CO_2_]. The trees were estimated from the elemental flows between plant parts in “Koshihikari”. Hierarchic clustering was calculated by group average method based on Euclidean distance.
(PDF)

S1 File Original data of Fig 1A. (XLSX)

S2 File Original data of Figs 1B and 2A (Relative contents of 18 elements). (XLSX)

S3 File Original data of Table 2 (Microarray analysis). (XLSX)
