# Supplementary material for: How elevated CO2 affects our nutrition in rice, and how we can deal with it
Source: PLoS One. 2019 Mar 5;14(3):e0212840. doi: 10.1371/journal.pone.0212840 (PMC6400444; doi:10.1371/journal.pone.0212840)
Supplement: S1 Table — Source: United Nations Food and Agriculture Organization food balance sheets and 2011 United Nations estimated population. (http://faostat3.fao.org/browse/FB/*/E) Under elevated [CO2], countries shown on a red background were estimated the protein supplies at the below level of hunger. The standard of hunger (52.5g/day) is calculated by the below; Standard in cal. by WHO/FAO 2,100 kcal per a day (ref. 1) Standard in protein by UNHCR, UNICEF, WFP and WHO <10% of cal. (ref. 2) Cal. per protein 4 kcal/g. (PDF) [file pone.0212840.s001.pdf]

S1 Table. Countries whose populations received protein from rice in 2011.

Countries	Population (in thousands)	Total (g $capita^{-1}$ day^{-1})	% Protein from Rice grain	Rice (g $capita^{-1}$ day^{-1})	Wheat (g $capita^{-1}$ day^{-1})	Protein supply under high CO_2 condition (Brown grain) (g $capita^{-1}$ day^{-1})	Protein supply under high CO_2 condition (Polished grain) (g $capita^{-1}$ day^{-1})	Protein supply from rice and wheat under high CO_2 condition (Brown grain) (g $capita^{-1}$ day^{-1})	Protein supply from rice and wheat under high CO_2 condition (Polished grain) (g $capita^{-1}$ day^{-1})
Tajikistan	7,814	52.6	2.5	1.3	28.6	52.5	52.4	48.6	48.6
Bangladesh	152,804	54.8	58.9	32.3	4.3	51.1	50.4	50.5	49.9
Yemen	23,299	57.0	5.1	2.9	27.4	56.7	56.6	53.0	52.9
Sierra Leone	5,862	56.0	31.3	17.5	2.8	54.0	53.6	53.6	53.3
Afghanistan	29,086	58.9	5.4	3.2	37.3	58.5	58.5	53.5	53.4
Sri Lanka	20,921	59.2	33.6	19.9	8.6	56.9	56.5	55.7	55.3
Thailand	66,558	58.7	33.0	19.4	2.4	56.5	56.1	56.1	55.8
Iraq	31,831	61.4	9.6	5.9	34.8	60.7	60.6	56.0	55.9
India	1,220,794	60.1	22.1	13.3	14.7	58.6	58.3	56.6	56.3
Philippines	95,036	60.2	33.9	20.4	3.0	57.8	57.4	57.4	57.0
Indonesia	243,740	61.1	39.6	24.2	4.5	58.3	57.8	57.7	57.2
Laos	6,518	63.4	53.0	33.6	0.4	59.5	58.9	59.5	58.8
Cambodia	14,600	63.2	49.1	31.0	0.5	59.6	59.0	59.6	58.9
Pakistan	176,099	64.7	3.7	2.4	23.6	64.4	64.4	61.2	61.2
Nepal	27,139	66.0	24.4	16.1	10.2	64.1	63.8	62.8	62.4
Syrian Arab Republic	21,809	78.3	2.4	1.9	35.5	78.1	78.0	73.3	73.2
Viet Nam	89,901	77.7	36.7	28.5	1.9	74.4	73.8	74.2	73.6
Malaysia	28,754	79.3	16.9	13.4	10.4	77.8	77.5	76.3	76.1
Libya	6,102	81.2	3.8	3.1	32.9	80.8	80.8	76.4	76.3
Myanmar	52,340	82.0	33.4	27.4	1.2	78.8	78.3	78.7	78.1
Jordan	6,730	83.8	4.7	3.9	36.0	83.4	83.3	78.5	78.4
Iran (Islamic Rep of)	75,400	86.5	6.2	5.4	36.0	85.9	85.8	81.0	80.9
Algeria	37,757	90.4	0.4	0.4	42.9	90.4	90.3	84.6	84.5
Japan	127,271	88.4	12.2	10.8	10.9	87.2	86.9	85.7	85.5
Morocco	32,051	95.6	0.1	0.1	42.0	95.6	95.6	89.9	89.9
China (Except Hong Kong and Macao)	1,368,340	95.3	15.9	15.2	17.7	93.5	93.2	91.2	90.9
Tunisia	10,750	98.0	0.3	0.3	49.3	98.0	98.0	91.3	91.3
Egypt	79,372	102.5	7.7	7.9	35.4	101.6	101.4	96.8	96.6
Total of population that over 50% protein is supplied from Rice	37,899								
Total of population that over 30% protein is supplied from Rice	342,765								
Total of population that over 15% protein is supplied from Rice	494,302								

Source: United Nations Food and Agriculture Organization food balance sheets and 2011 United Nations estimated population. (http://faostat3.fao.org/browse/FB/*/E)

Under elevated [CO_2], countries shown on a red background were estimated the protein supplies at the below level of hunger.

The standard of hunger (52.5g/day) is calculated by the below;

Standard in cal. by WHO/FAO	2,100 kcal per a day (ref. 1)
Standard in protein by UNHCR, UNICEF, WFP and WHO	<10% of cal. (ref. 2)
Cal. per protein	4 kcal/g

Reference;

1. Joint WHO/FAO Expert Consultation on Diet, Nutrition and the Prevention of Chronic Diseases (2002)

2 UNHCR, UNICEF, WFP, WHO Food and Nutrition Needs in Emergencies (2002)
