# Supplementary material for: How elevated CO2 affects our nutrition in rice, and how we can deal with it
Source: PLoS One. 2019 Mar 5;14(3):e0212840. doi: 10.1371/journal.pone.0212840 (PMC6400444; doi:10.1371/journal.pone.0212840)
Supplement: S2 Table — Source: USDA National Nutrient Database for Standard Reference (long and medium grain rice) (http://ods.od.nih.gov/). Standard tables of food composition in Japan, Fifth Revised and Enlarged Edition—2005 - (short grain rice). (http://www.mext.go.jp/b_menu/shingi/gijyutu/gijyutu3/toushin/05031802/002/001.pdf) (PDF) [file pone.0212840.s002.pdf]

S2 Table. The mineral content of brown and polished grain in rice (Value per 100g).

	Unit	long grain (brown)	long grain (polished)	medium grain (brown)	medium grain (polished)	short grain (brown)	short grain (polished)
Nitrogen, N	mg	7.94	7.13	7.5	6.61	6.8	6.1
Calcium, Ca	mg	52	28	33	9	9	5
Iron, Fe	mg	1.48	0.8	1.8	0.8	2.1	0.8
Magnesium, Mg	mg	46	25	143	35	110	23
Phosphorus, P	mg	213	115	264	108	290	94
Potassium, K	mg	213	115	268	86	230	88
Sodium, Na	mg	9	5	4	1	1	1
Zinc, Zn	mg	2.02	1.09	2.02	1.16	1.8	1.4

Source: USDA National Nutrient Database for Standard Reference (long and medium grain rice)
(http://ods.od.nih.gov/)

Standard tables of food composition in Japan, Fifth Revised and Enlarged Edition - 2005 - (short grain rice)
(http://www.mext.go.jp/b_menu/shingi/gijyutu/gijyutu3/toushin/05031802/002/001.pdf)
