# Supplementary material for: How elevated CO2 affects our nutrition in rice, and how we can deal with it
Source: PLoS One. 2019 Mar 5;14(3):e0212840. doi: 10.1371/journal.pone.0212840 (PMC6400444; doi:10.1371/journal.pone.0212840)
Supplement: S3 Table — The values indicate the ratio of the mineral content in polished grain to content in brown grain (= 100). (PDF) [file pone.0212840.s003.pdf]

S3 Table. The ratio in mineral content between brown and polished grain in rice.

ratio	Unit	long grain	medium grain	short grain	Average
Nitrogen, N	(%)	89.8	88.1	89.7	89.2
Calcium, Ca	(%)	53.8	27.3	55.6	45.6
Iron, Fe	(%)	54.1	44.4	38.1	45.5
Magnesium, Mg	(%)	54.3	24.5	20.9	33.2
Phosphorus, P	(%)	54.0	40.9	32.4	42.4
Potassium, K	(%)	54.0	32.1	38.3	41.4
Sodium, Na	(%)	55.6	25.0	100.0	60.2
Zinc, Zn	(%)	54.0	57.4	77.8	63.1

The values indicate the ratio of the mineral content in polished grain to content in brown grain (=100).
