# Supplementary material for: How elevated CO2 affects our nutrition in rice, and how we can deal with it
Source: PLoS One. 2019 Mar 5;14(3):e0212840. doi: 10.1371/journal.pone.0212840 (PMC6400444; doi:10.1371/journal.pone.0212840)
Supplement: S4 Table — ND means no data. ***, **, * Significant at P<0.001, 0.01, 0.05. In plant species, B. means Brassica. (PDF) [file pone.0212840.s004.pdf]

S4 Table. Analysis of elevated CO_2 effects on elemental contents in leaf vegetables.

Plant species	Plant type	S	K	Ca	P	Mg	Zn	Fe	Mn	Cu	Refernce
B. rapa var. *previridis* (rakuten)	C_3	101.79	92.10	84.37	78.20	85.91	101.37	91.51	82.46	87.89	new data
B. rapa var. *previridis* (kahoku)	C_3	105.73	92.17	67.36	76.85	83.52	87.82	86.95	62.58	90.33	new data
B. rapa var. *chinensis*	C_3	134.63	90.51	63.52	92.63	75.03	89.43	87.70	86.54	91.57	new data
B. oleracea var. *capitata*	C_3	92.72	108.78	76.12	94.86	92.35	101.85	91.08	92.12	55.54	new data
Beta vulgaris var. *cilia*	C_3	108.72	95.89	72.84	85.64	84.20	95.12	89.31	80.93	81.34	new data
Apium graveolens var. *dulce*	C_3	ND	91.52	97.66	94.21	90.00	ND	ND	ND	ND	Tremblay et al, 1988
B. oleracea var. *alboglabra*	C_3	95.29	ND	ND	ND	ND	ND	ND	ND	ND	La et al., 2009
Spinacia oleracea	C_3	ND	ND	104.12	ND	39.29	ND	44.62	ND	ND	Jain et al., 2007
Ave.		106.48	95.16	80.86	87.07	78.61	95.12	81.86	80.93	81.34	
P value (ZTEST)		0.2920	0.0867	0.0009	0.0001	0.0019	0.0943	0.0154	0.0001	0.0052	
				***	***	**		*	***	**	

ND means no data. ***,**,* Significant at $P<0.001$, 0.01, 0.05

In plant species, B. means Brassica.

Reference;

1. Jain, V., Pal, M., Raj, A. and Khetarpal, S. Photosynthesis and nutrient composition of spinach and fenugreek grown. under elevated carbon dioxide concentration. *Biol. Plant* 51, 559–562 (2007).

2. La, G.X., Fang, P., Teng, Y.B., Li, Y.J. and Lin, X.Y. Effect of CO_2 enrichment on the glucosinolate contents under different nitrogen levels in bolting stem of Chinese kale (*Brassica alboglabra* L.) *J. Zhejiang Univ. Sci. B* 10, 454–464 (2009).

3. Tremblay, N., Yelle, S. and Gosselin, A. Effects of CO_2 enrichment, nitrogen and phosphorus fertilization during the nursery period on mineral composition of celery. *J. Plant Nutr.* 11, 37–49 (1988).
