# Supplementary material for: How elevated CO2 affects our nutrition in rice, and how we can deal with it
Source: PLoS One. 2019 Mar 5;14(3):e0212840. doi: 10.1371/journal.pone.0212840 (PMC6400444; doi:10.1371/journal.pone.0212840)
Supplement: S1 Fig — Actin (Act1) was used as the control. Data are presented as mean ± s.d. (n = 3). Student’s t tests were used to calculate P values. (PDF) [file pone.0212840.s005.pdf]

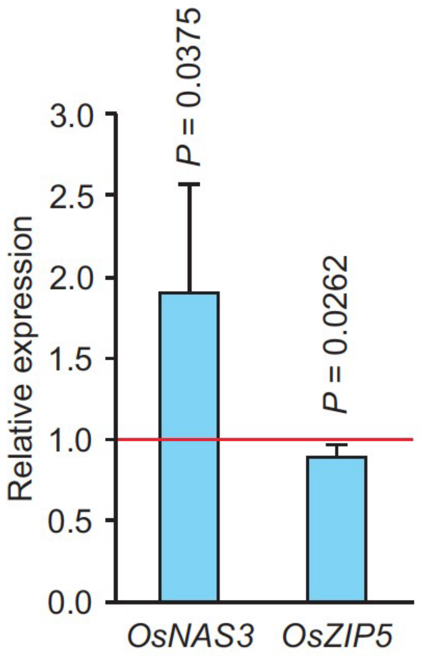

S1 Fig. Expression change of *OsNAS3* (Os07g0689600) and *OsZIP5* (Os05g0472700) under elevated [CO_2] condition relative to ambient [CO_2]. *Actin* (*Act1*) was used as the control. Data are presented as mean ± s.d. (n = 3). Student's t-tests were used to calculate P values.
