# Supplementary material for: How elevated CO2 affects our nutrition in rice, and how we can deal with it
Source: PLoS One. 2019 Mar 5;14(3):e0212840. doi: 10.1371/journal.pone.0212840 (PMC6400444; doi:10.1371/journal.pone.0212840)
Supplement: S2 Fig — The trees were estimated from the elemental flows between plant parts in “Koshihikari”. Hierarchic clustering was calculated by group average method based on Euclidean distance. (PDF) [file pone.0212840.s006.pdf]

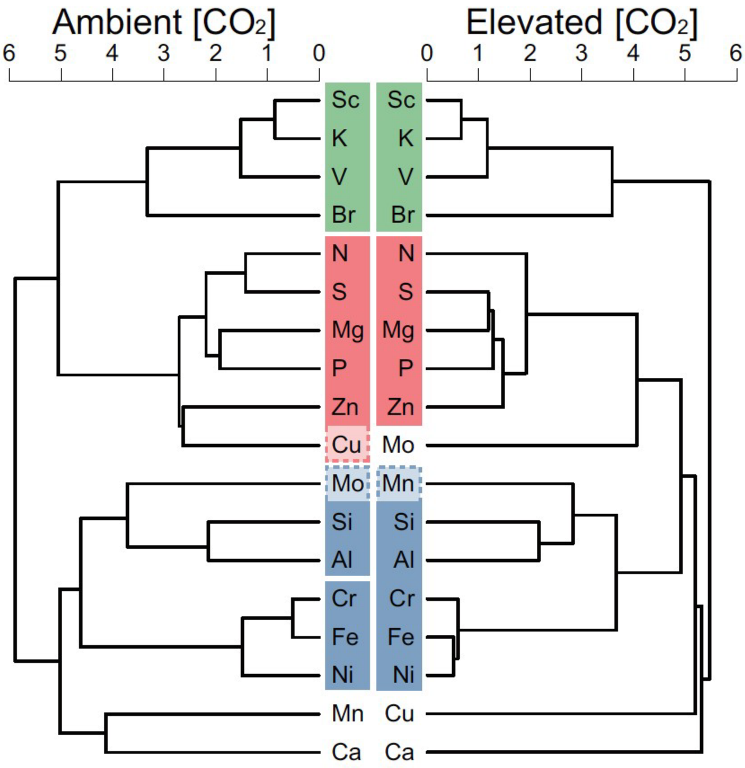

S2 Fig. Dendrogram for elemental flows in elevated [CO_2] and ambient [CO_2]. The tree were estimated from the elemental flows between plant parts in “Koshihikari”. Hierarchic clustering was calculated by group average method based on Euclidean distance.
